# Supplementary material for: Energy stress-induced lncRNA FILNC1 represses c-Myc-mediated energy metabolism and inhibits renal tumor development
Source: Nat Commun. 2017 Oct 4;8:783. doi: 10.1038/s41467-017-00902-z (PMC5627275; doi:10.1038/s41467-017-00902-z)
Supplement: Supplementary file 1 — Supplementary Information [file 41467_2017_902_MOESM1_ESM.pdf]

Description of Supplementary Files

File Name: Supplementary Information
Description: Supplementary Figures and Supplementary Methods

File Name: Supplementary Data 1
Description: The list of FILNC1-interacting proteins under 1 mM glucose condition identified by mass spectrometry analysis.

File Name: Peer Review File

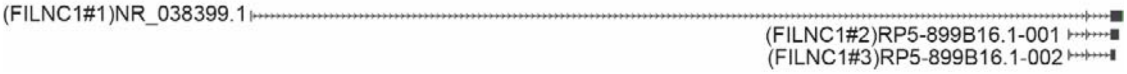

Supplementary Figure 1. The schematic diagram of the genomic region of *FILNC1* with different splicing isoforms. Arrows and black boxes represent the direction of transcription and exons respectively.

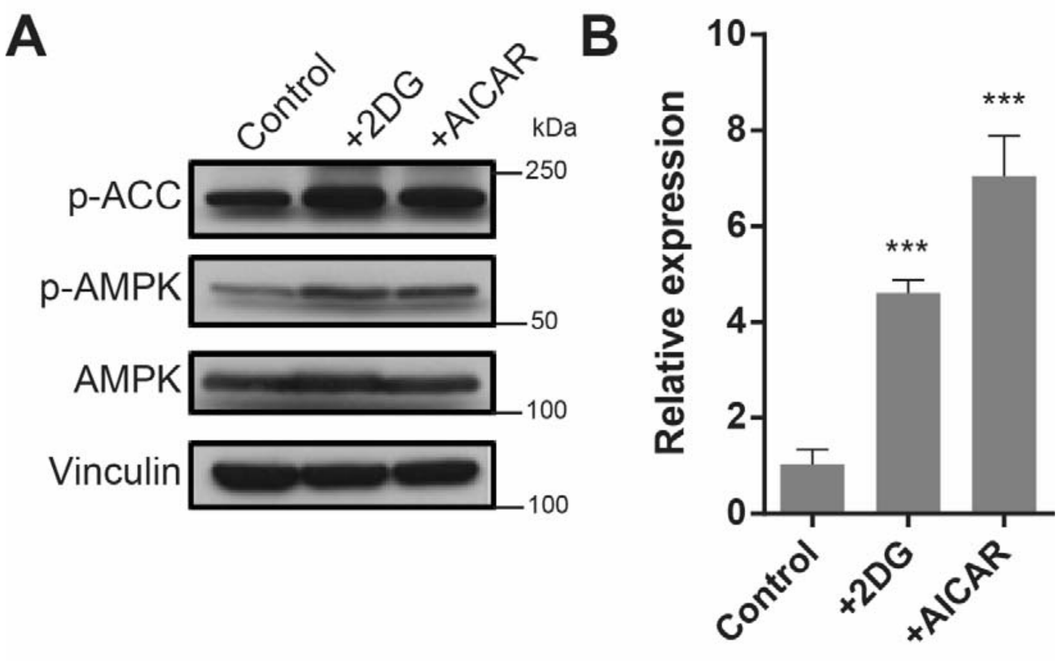

Supplementary Figure 2. *FILNC1* is up-regulated in renal cancer cells when treated with 2DG or AICAR. (A) 786-O cells were treated with 5 mM 2-DG or 500 nM AICAR for 24 hours and then were subjected to Western blot. **(B)** Bar graph shows the relative expression changes of *FILNC1* by real-time PCR in 786-O cells that had been treated with 5 mM 2DG or 500 nM AICAR for 24 hours. Values represent mean ± s.d. from three independent experiments, two-tailed Student's t-test. ***: $P < 0.001$.

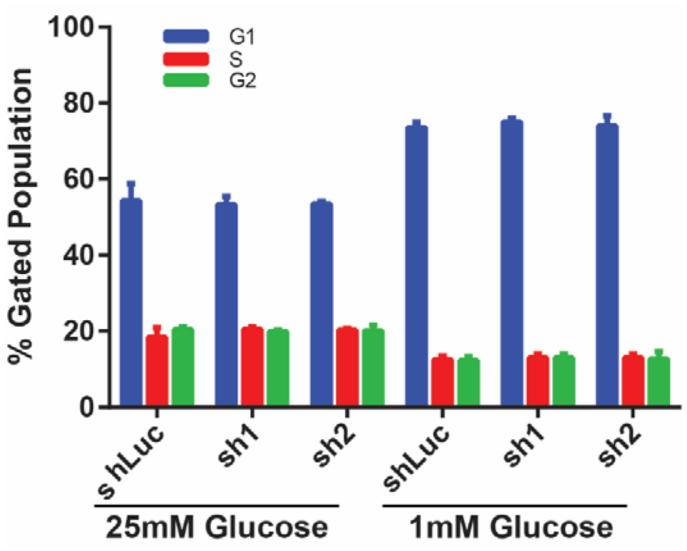

Supplementary Figure 3. ***FILNC1*** **deficiency does not affect the cell cycle.** 786-O cells infected with control shRNA or *FILNC1* shRNAs were cultured in 25 or 1 mM glucose-containing medium for 24 hours, and then subjected to cell cycle analysis by flow cytometry. Values represent mean ± s.d. from three independent experiments.

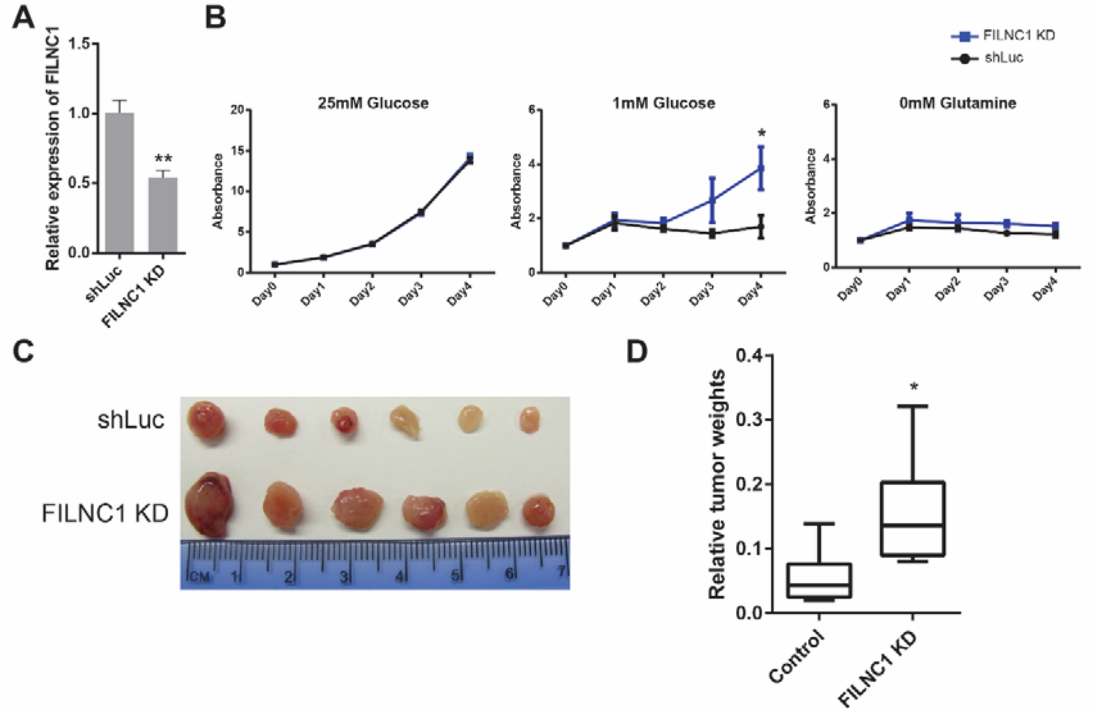

Supplementary Figure 4. *FILNC1* deficiency protects renal cancer from cell death under energy stress and promotes renal tumor development. (A) Bar graph shows *FILNC1* shRNA-mediated knockdown efficiency in UMRC2 cells. **(B)** UMRC2 cells infected with either control shRNA or *FILNC1* shRNA were cultured in various medium for different days as indicated, and then subjected to cell proliferation analysis. **(C-D)** Xenograft tumour images **(C)** and weights **(D,** mean ± s.d, n=6 xenograft tumors, two-tailed Student's t-test**)** of UMRC2 cells infected with either control shRNA or *FILNC1* shRNA. All values, unless otherwise noted, represent mean ± s.d. from three independent experiments, two-tailed Student's t-test. *: P < 0.05.

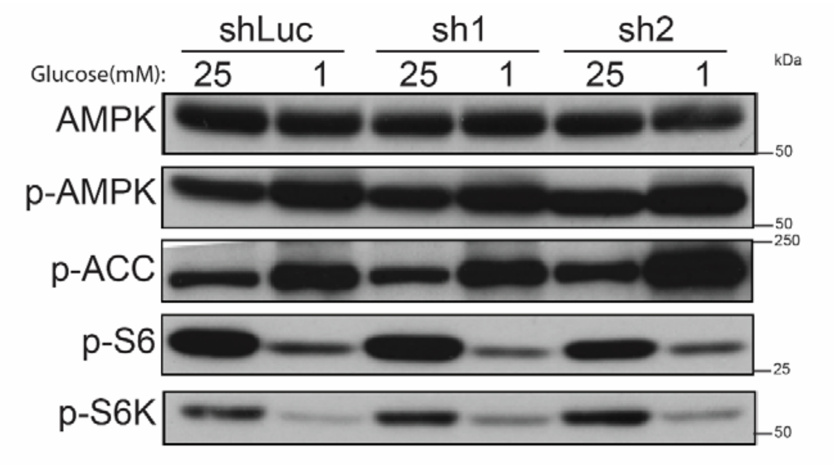

Supplementary Figure 5. *FILNC1* deficiency does not affect AMPK activation or mTORC1 inactivation under glucose starvation. Control shRNA or *FILNC1* shRNA-infected 786-O cells were cultured in 25 or 1 mM glucose-containing medium for 24 hours, then subjected to Western blotting analysis as indicated.

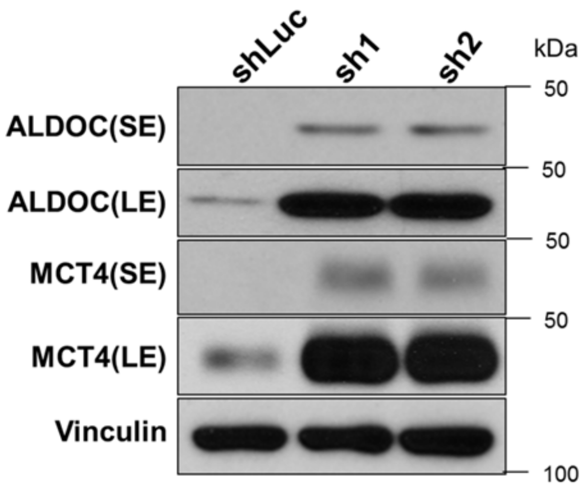

Supplementary Figure 6. *FILNC1* knockdown increases ALDOC and MCT4 protein level. Control shRNA or *FILNC1* shRNA-infected 786-O cells were cultured 1 mM glucose-containing medium for 24 hours, then subjected to Western blotting analysis. SE, short exposure; LE, long exposure.

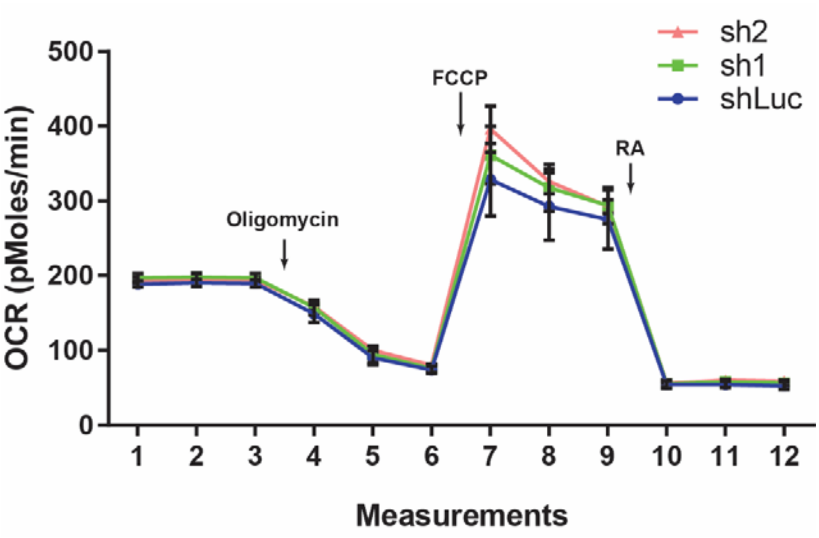

Supplementary Figure 7. ***FILNC1* deficiency does not affect oxygen consumption rate under glucose starvation.** Oxygen consumption rate of Control and *FILNC1* knock-down 786-O cells was measured in the XF96 Analyzer. All values represent mean ± s.d. from three independent experiments.

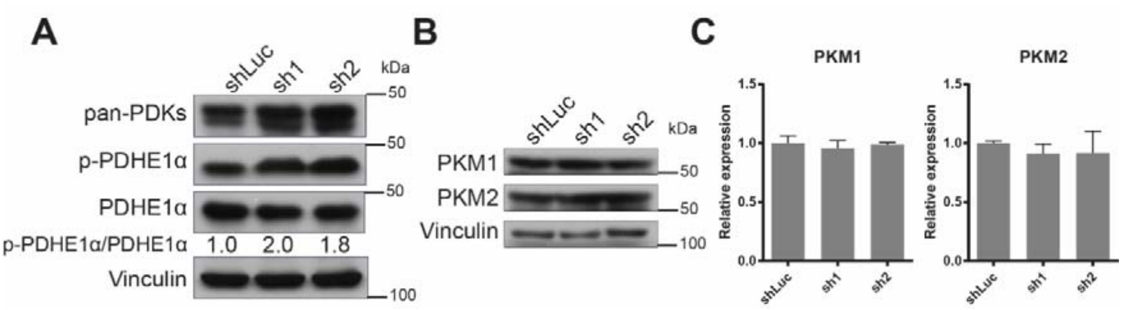

Supplementary Figure 8. *FILNC1* knockdown increases PDK protein level and PDH phosphorylation, but does not affect PKM1/2 isoform switch. (A-B) Control shRNA or *FILNC1* shRNA-infected 786-O cells were cultured 1 mM glucose-containing medium for 24 hours, then subjected to Western blotting analysis. **(C)** Real-time PCR analysis of PKM1 and PKM2 in control shRNA or *FILNC1* shRNA-infected 786-O cells under low glucose condition. All values represent mean ± s.d. from three independent experiments.

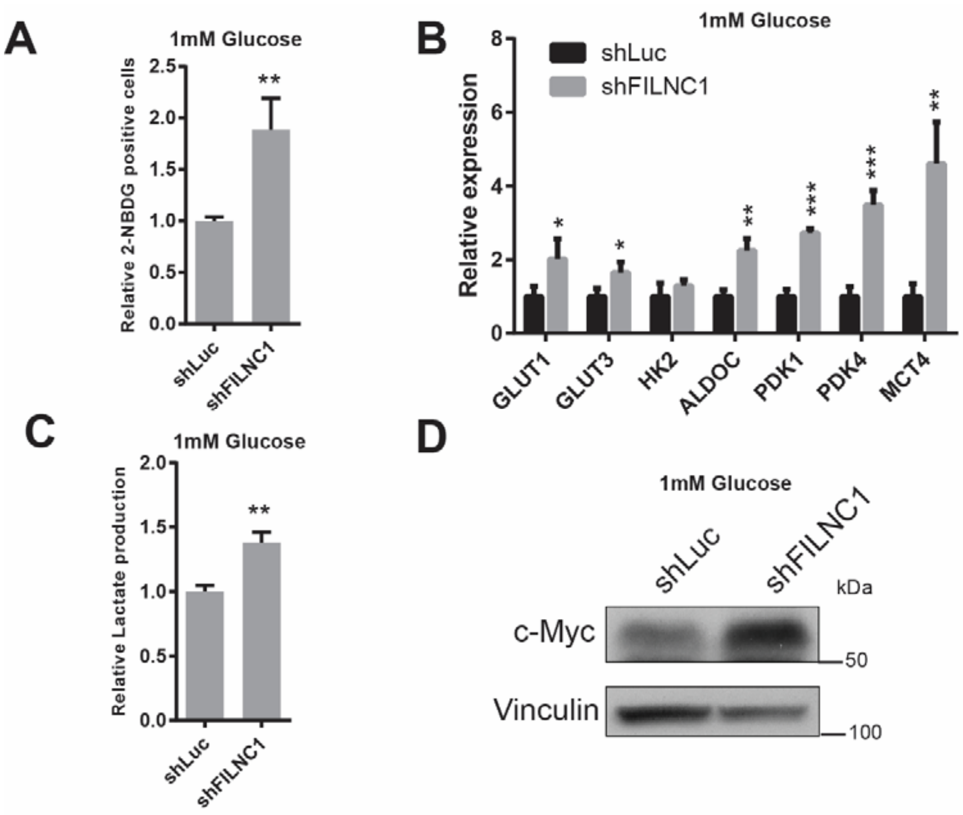

Supplementary Figure 9. *FILNC1* deficiency increases c-Myc protein level and glucose metabolism genes, and promotes glucose uptake and lactate production in UMRC2 cells. (**A-C**) UMRC2 cells infected with either control shRNA or *FILNC1* shRNA were cultured in 25 or 1 mM glucose-containing medium for 48 hours, and then subjected to various analyses to measure the expression levels of genes involved in glucose metabolism by real-time PCR (**A**), glucose uptake **(B)** and lactate production **(C)**. All values represent mean ± s.d. from three independent experiments, two-tailed Student's t-test. *: P < 0.05; **: P < 0.01; ***: P < 0.001. **(D)** Western blotting analysis to measure c-Myc protein in UMRC2 cells infected with control shRNA or *FILNC1* shRNA which had been cultured in 1 mM glucose-containing medium for 24 hours.

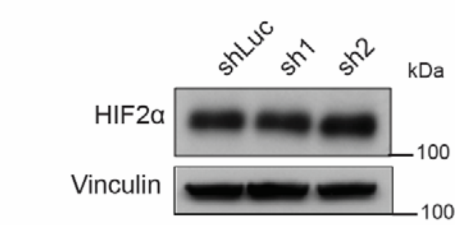

Supplementary Figure 10. FILNC1 knockdown did not affect HIF2α protein levels. Control or *FILNC1* shRNA-infected 786-O cells were cultured in 1 mM glucose-containing medium for 24 hours, then subjected to Western blotting analysis to measure HIF2α protein.

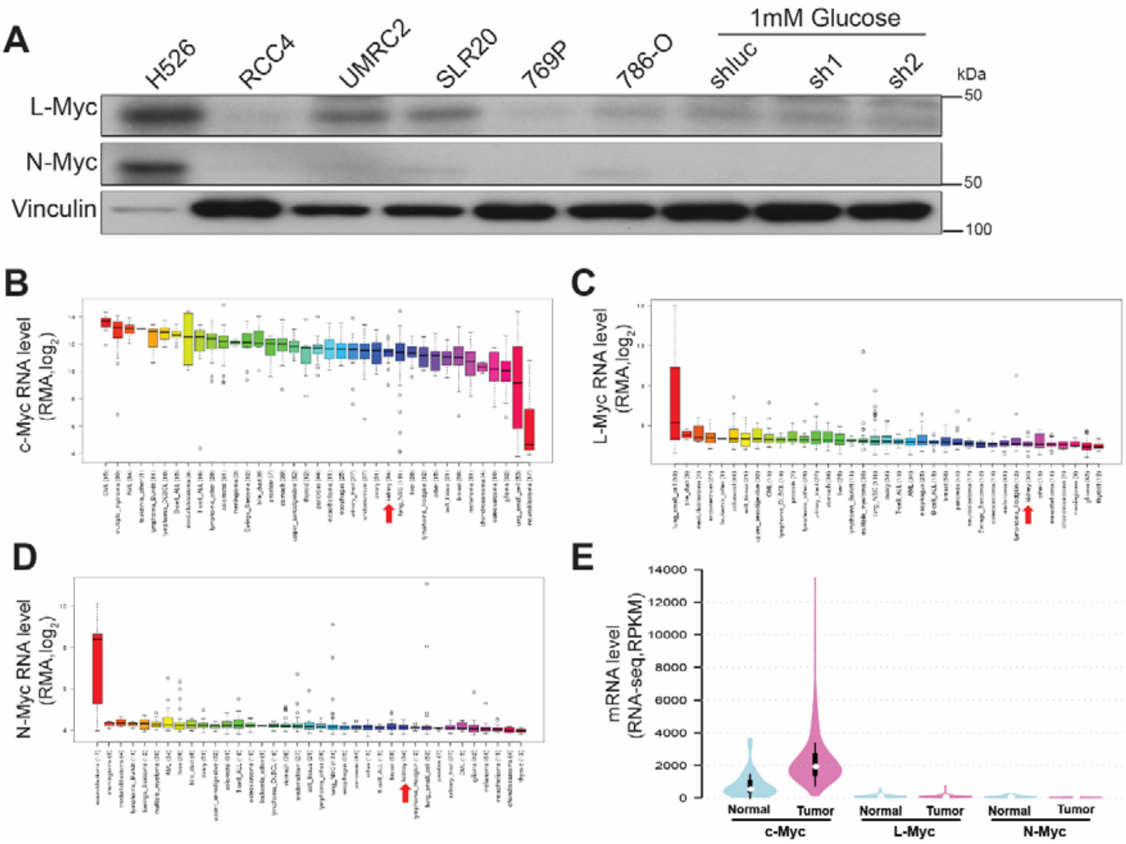

Supplementary Figure 11. L-Myc and N-Myc expression in renal cancer cell lines and tumors. (A) The protein levels of L- and N-Myc in kidney cancer cells and control shRNA or *FILNC1* shRNA-infected 786-O cells under glucose starvation condition. H526 lung cancer cell was used as a positive control. **(B-D)** The mRNA expression levels of *c-Myc*, *L-Myc*, and *N-Myc* in various cancer cell lines. Red arrows point to renal cancer cells. Expression data were obtained from CCLE expression datasets. **(E)** The mRNA expression levels of *c-Myc*, *L-Myc*, and *N-Myc* in renal cancer and normal kidneys. Expression data were obtained from TCGA ccRRCC RNA-seq datasets.

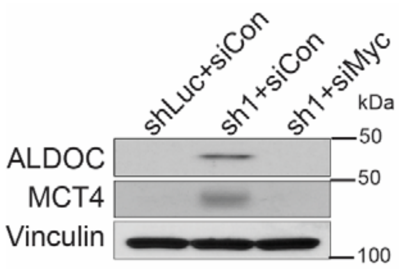

Supplementary Figure 12. Knocking down c-Myc in *FILNC1* deficient 786-O cells represses the ALDOC and MCT4 protein levels. Control shRNA or *FILNC1* shRNA-infected 786-O cells were transfected with c-Myc siRNA. The cells were cultured in 1 mM glucose-containing medium for 24 hours, and c-Myc protein level was detected by Western blotting.

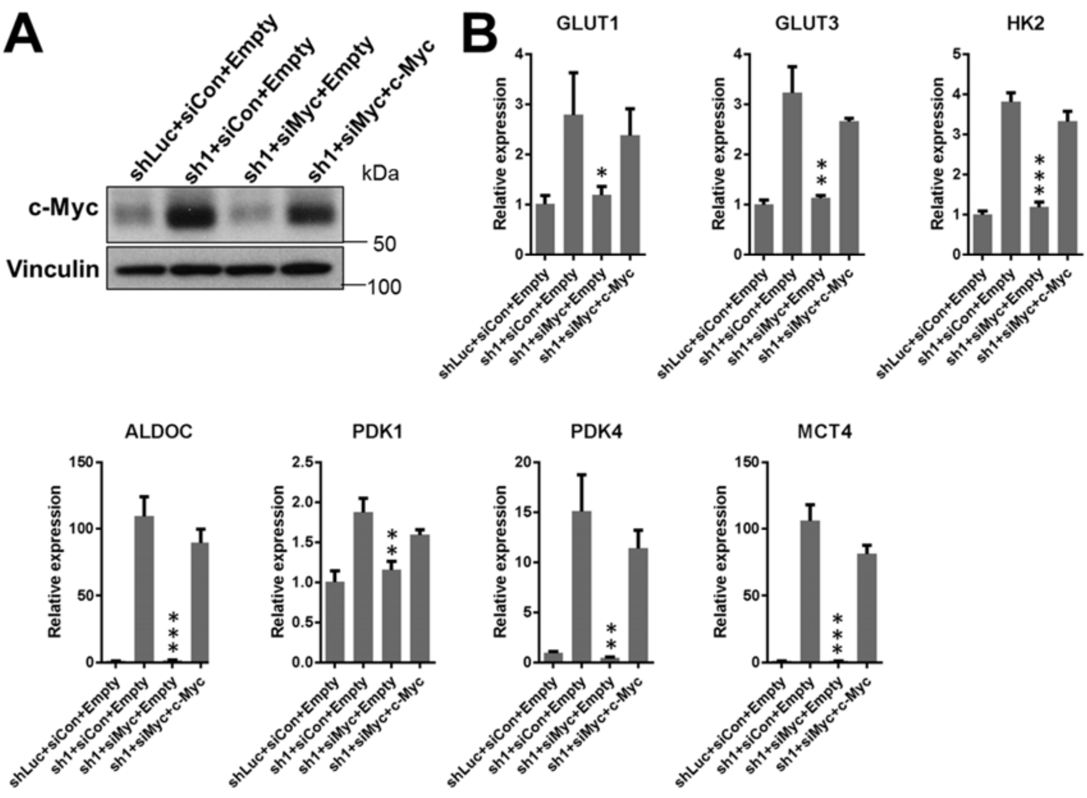

Supplementary Figure 13. c-Myc restoration normalized the downregulation of expression levels of glucose metabolism genes caused by c-Myc knockdown in 786-O cells. c-Myc was re-expressed in *FILNC1*/*c-Myc* double knockdown cells. The cells were cultured in 1 mM glucose-containing medium for 24 hours, and c-Myc protein level was detected by Western blotting (**A**). The expression levels of genes involved in glucose metabolism were measured by real-time PCR (**B**). All values represent mean ± s.d. from three independent experiments, two-tailed Student's t-test. *: $P < 0.05$; **: $P < 0.01$; ***: $P < 0.001$.

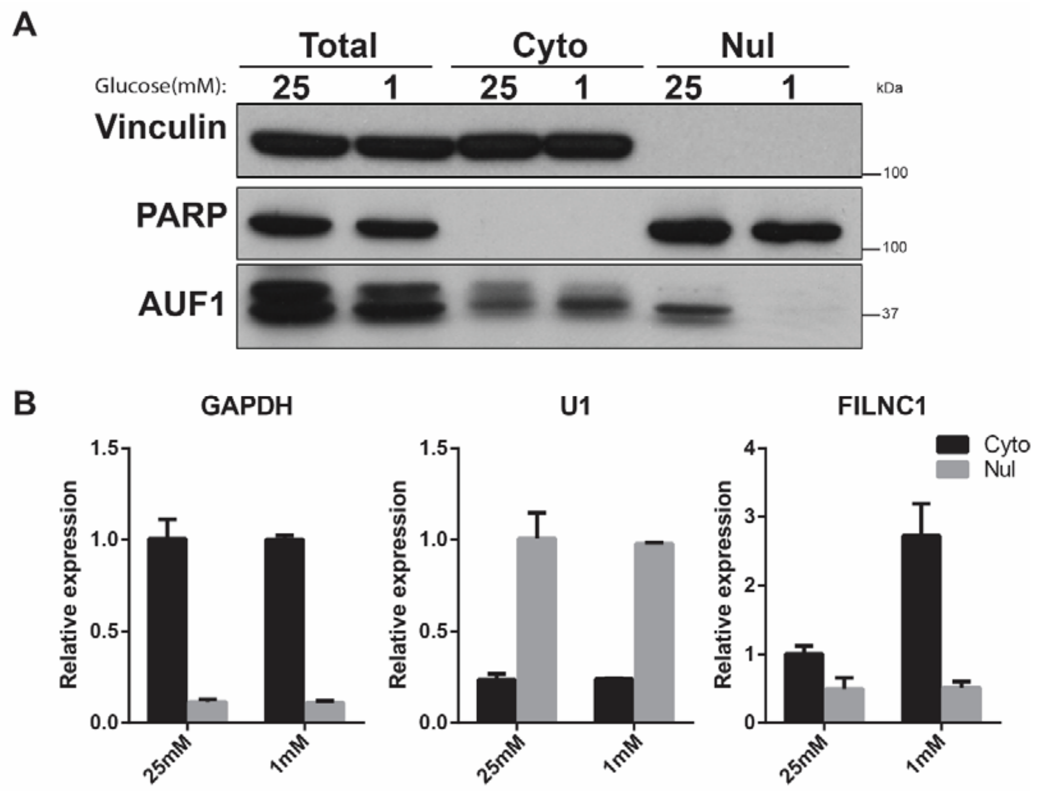

Supplementary Figure 14. Glucose starvation regulates AUF1 and FILNC1 subcellular localization. 786-O cells were cultured in 20 mM or 1 mM glucose for 24 hours, then subjected to nucleus (nul)/cytoplasm (cyt) fractionation analysis and Western blotting **(A)** or real-time PCR experiments **(B)**. Vinculin and PARP serve as markers of cytoplasm and nucleus in Western blotting. GAPDH and U1 serve as markers of cytoplasm and nucleus in real-time PCR. All values, unless otherwise noted, represent mean ± s.d. from three independent experiments.

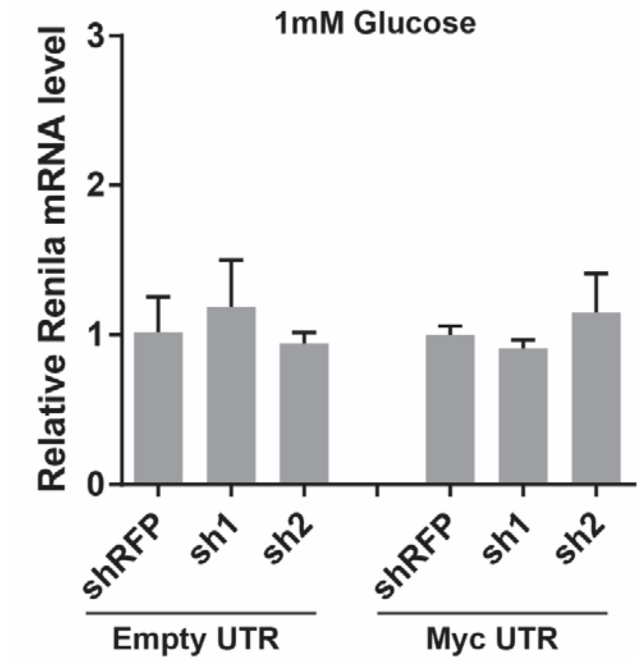

Supplementary Figure 15. *FILNC1* deficiency does not affect luciferase reporter mRNA level. Renilla luciferase mRNA level of a reporter fused to a c-Myc or empty 3′ UTR in 786-O cells that had been cultured in 1 mM glucose-containing medium for 24 hours. mRNA levels were normalized using Firefly luciferase mRNA levels. All values mean ± s.d. from three independent experiments.

Raw C_t Values

	786-O			769P+*FILNC1*		
GAPDH	15.04	15.8	15.77	15.68	15.51	15.7
FILNC1	24.21	24.71	24.04	22.85	21.95	23.09

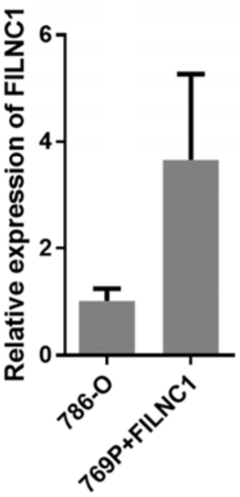

Supplementary Figure 16. Comparison of *FILNC1* expression in 786-O and *FILNC1* over-expressed 769P cells under glucose starvation condition. The raw C_t values and relative expression levels of endogenous *FILNC1* in 786-O cells and over-expressed *FILNC1* in 769P cells cultured in 1 mM glucose-containing medium for 24 hours were measured by real-time PCR. All values mean ± s.d. from three independent experiments.

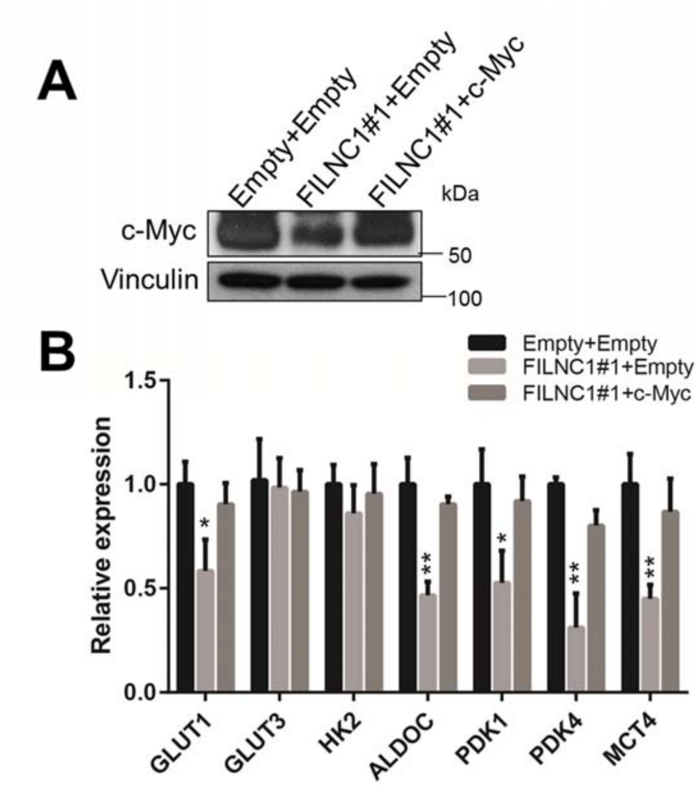

Supplementary Figure 17. c-Myc restoration normalized the downregulation of expression levels of glucose metabolism genes caused by *FILNC1* over-expression in 769P cells. c-Myc was re-expressed in *FILNC1* over-expressed 769P cells. The cells were cultured in 1 mM glucose-containing medium for 24 hours, and c-Myc protein level was detected by Western blotting (**A**). The expression levels of genes involved in glucose metabolism were measured by real-time PCR (**B**). All values represent mean ± s.d. from three independent experiments, two-tailed Student's t-test. *: P < 0.05; **: P < 0.01; ***: P < 0.001.

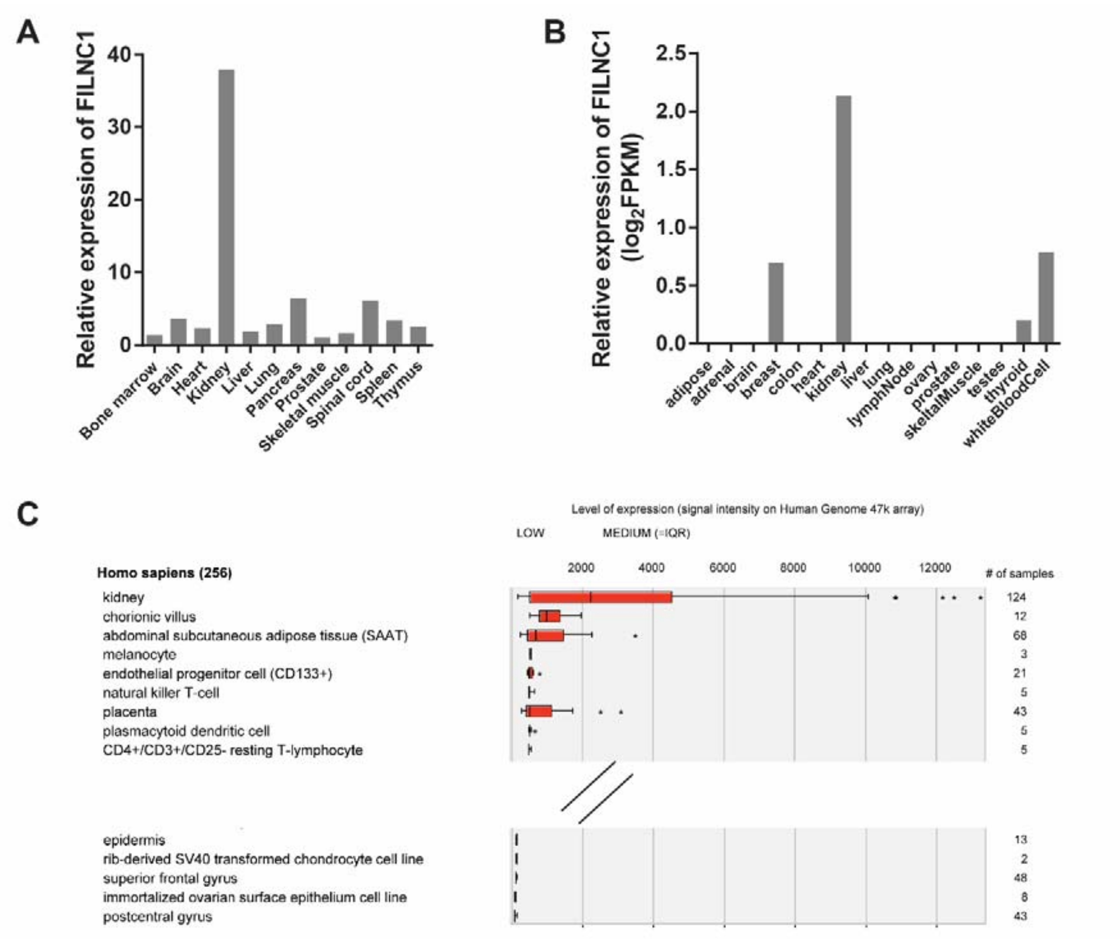

Supplementary Figure 18. *FILNC1* is highly expressed in kidney. *FILNC1* expression levels in human tissues were examined from public data set GSE803 (**A**), Human lincRNA Catalog http://www.broadinstitute.org/genome_bio/human_lincrnas/ **(B),** or GENEVESTIGATOR **(C).**

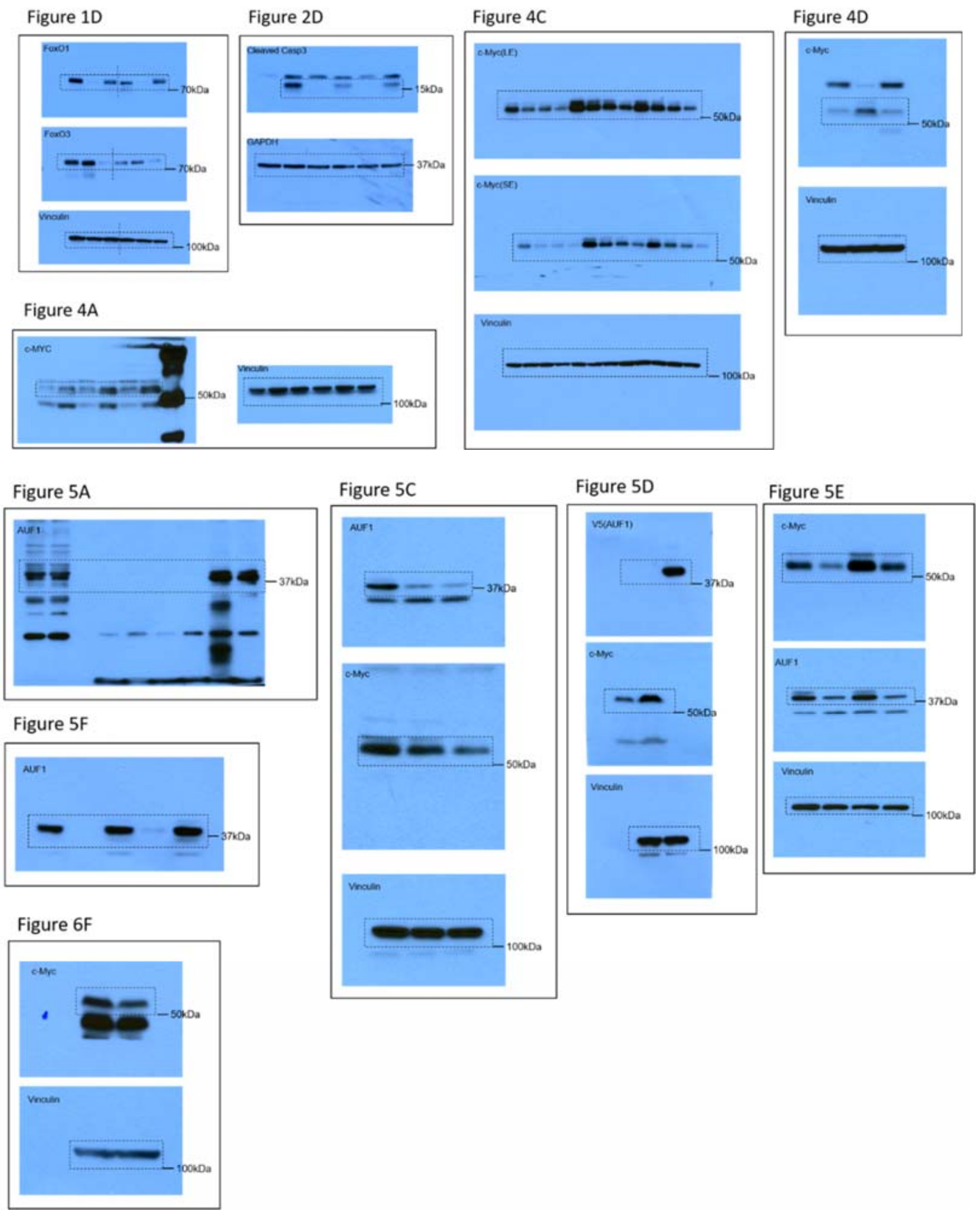

Supplementary Figure 19. Raw western blot images corresponding with main figures. Portions of blots presented in the main paper were boxed.

Supplementary Methods

The sequences for all primer used are listed below:

Primers for ChIP:

Site1-Forward: 5'- GCTTTATGCAGTGAATGAGCTTTA-3'

Site1-Reverse: 5'- AGATATCAAAGAATTTCCCCATGA-3'

Site2-Forward: 5'- CTGTGCTTCTCCCTTAAAAACAAT-3'

Site2-Reverse: 5'- TTCAATAGACACTTGCAACAGTGA-3'

Primers for shRNA:

shLuc:5'-
CCGGGATTTCGAGTCGTCTTAATCTCGAGATTAAGACGACTCGAAATCTTTTT
G-3'

shRFP: 5'-CCGGTGCTAAGGAGTTTGGAGACAACTCGAGTTGTCTCCAAAC

TCCTTAGCATTTTTG-3'

FILNC1-shRNA1:5'-
CCGGACACGTTGGACTTGGAATAATCTCGAGATTATTCCAAGTCCAACGTGTT
TTTTG-3'

FILNC1-shRNA2:5'-
CCGGGATTCCAATCTGTGCCTAAATCTCGAGATTTAGGCACAGATTGGAATCT
TTTTG-3'

AUF1-shRNA1:5'-
CCGGTCGAAGGAACAATATCAGCAACTCGAGTTGCTGATATTGTTCCTTCGAT
TTTTG-3'

AUF1-shRNA2:5’-
CCGGTCGAAGGAACAATATCAGCAACTCGAGTTGCTGATATTGTTCCTTCGATTTTT-3’

Real time PCR primers:

FILNC1-Forward: 5’- CCACTCAGTTCTTCATGCTTGT-3’

FILNC1-Reverse: 5’- GAAAGGCTGTTTGTTTTGCTGT-3’

GAPDH-Forward: 5’- CCATGGGGAAGGTGAAGGTC-3’

GAPDH -Reverse: 5’-GAAGGGGTCATTGATGGCAAC-3’

U1-Forward: 5’-TCCCAGGGCGAGGCTTATCCATT-3’

U1-Reverse: 5’-GAACGCAGTCCCCCACTACCACAAAT-3’

c-Myc-Forward: 5’- CAGCTGCTTAGACGCTGGATTT -3’

c-Myc-Reverse: 5’- ACCGAGTCGTAGTCGAGGTCAT -3’

GLUT1-Forward: 5’- ATGATGCGGGAGAAGAAGGTC-3’

GLUT1-Reverse: 5’- TCGTGGAGTAATAGAAGACAGCG-3’

GLUT3-Forward: 5’- TTGAACACCTGCATCCTTGA-3’

GLUT3-Reverse: 5’- GACAGCCCATCATCATTTCC-3’

HK2-Forward: 5’- AGCCCTTTCTCCATCTCCTT-3’

HK2-Reverse: 5’- AACCATGACCAAGTGCAGAA-3’

ALDOC-Forward: 5’- CAGGGCAATGTCAGACAACT-3’

ALDOC -Reverse: 5’- GGCTGCGGCTGCTAACT-3’

PDK1-Forward: 5’- ATTTTCCTCAAAGGAACGCC-3’

PDK1-Reverse: 5’- CAACAGAGGTGTTTACCCCC-3’

PDK4-Forward: 5’- CACGATGTGAATTGGTTGGT-3’

PDK4-Reverse: 5’- TGCCTTTGAGTGTTCAAGGA-3’

MCT4-Forward: 5’- AAGAGCCATTGTATGTCTGGG-3’

MCT4-Reverse: 5’- GGGACTTGCCAGTTTCTTTG-3’

PKM1-Forward: 5’- ACCGCAAGCTGTTTGAAGAA-3’

PKM1-Reverse: 5’- TCCATGAGGTCTGTGGAGTG-3’

PKM2-Forward: 5’- GAGGCCTCCTTCAAGTGCT-3’

PKM2-Reverse: 5’- CCAGACTTGGTGAGGACGAT-3’

Renilla Luciferase-Forward: 5’- AACTGGAGCCTGAGGAGTTC-3’

Renilla Luciferase-Reverse: 5’- TAGCTCCCTCGACAATAGCG-3’

Firefly Luciferase-Forward: 5’- CATTCTTCGAGGCCAAGGTG-3’

Firefly Luciferase-Reverse: 5’- CCACGATGAAGAAGTGCTCG-3’

Primers for RNA pull down assay:

FILNC1-Forward: 5’-
TAATACGACTCACTATAGGGGATTTGCAGAAAGAGCCAACA-3’

FILNC1-Reverse: 5’-AAACAAGATAGTTTATTTTTCTTGCAC-3’

NBR2-Forward: 5’-TAATACGACTCACTATAGGGAGGGGTCCAGTTGCGGCTTAT
-3’

NBR2-Reverse: 5’-AGTTTACTTACTATTGCTGA -3’

Myc 3’ UTR-Forward: 5’-
TAATACGACTCACTATAGGGGGAAAAGTAAGGAAAACGATTCCT -3’

Myc 3’ UTR -Reverse: 5’- AAGATTTGGCTCAATGATATATTTGCCAG -3’

Primers for FILNC1 clone:

OE-Forward: 5'-ACGCCTCGAGGATTTGCAGAAAGAGCCAACA-3'

OE- Reverse: 5'-ACGCGGATCCAAACAAGATAGTTTATTTTTCTTGCAC-3'
